# DNA Methylation of Specific CpG Sites in the Promoter Region Regulates the Transcription of the Mouse Oxytocin Receptor

**DOI:** 10.1371/journal.pone.0056869

**Published:** 2013-02-18

**Authors:** Shimrat Mamrut, Hala Harony, Rapita Sood, Hadar Shahar-Gold, Harold Gainer, Yi-Jun Shi, Liza Barki-Harrington, Shlomo Wagner

**Affiliations:** 1 Department of Neurobiology, University of Haifa, Haifa, Israel; 2 Department of Human Biology, University of Haifa, Haifa, Israel; 3 Laboratory of Neurochemistry, National Institute of Neurological Disorders and Stroke, National Institutes of Health, Bethesda, Maryland, United States of America; CNRS UMR7275, France

## Abstract

Oxytocin is a peptide hormone, well known for its role in labor and suckling, and most recently for its involvement in mammalian social behavior. All central and peripheral actions of oxytocin are mediated through the oxytocin receptor, which is the product of a single gene. Transcription of the oxytocin receptor is subject to regulation by gonadal steroid hormones, and is profoundly elevated in the uterus and mammary glands during parturition. DNA methylation is a major epigenetic mechanism that regulates gene transcription, and has been linked to reduced expression of the oxytocin receptor in individuals with autism. Here, we hypothesized that transcription of the mouse oxytocin receptor is regulated by DNA methylation of specific sites in its promoter, in a tissue-specific manner. Hypothalamus-derived GT1-7, and mammary-derived 4T1 murine cell lines displayed negative correlations between oxytocin receptor transcription and methylation of the gene promoter, and demethylation caused a significant enhancement of oxytocin receptor transcription in 4T1 cells. Using a reporter gene assay, we showed that methylation of specific sites in the gene promoter, including an estrogen response element, significantly inhibits transcription. Furthermore, methylation of the oxytocin receptor promoter was found to be differentially correlated with oxytocin receptor expression in mammary glands and the uterus of virgin and post-partum mice, suggesting that it plays a distinct role in oxytocin receptor transcription among tissues and under different physiological conditions. Together, these results support the hypothesis that the expression of the mouse oxytocin receptor gene is epigenetically regulated by DNA methylation of its promoter.

## Introduction

Oxytocin (OT) is a nonapeptide, mostly known for its role in enhancing contractions of the uterus during labor and mediating milk release from mammary glands during suckling [1]. Recently, OT activity in the brain was shown to play an important role in mammalian social behavior [2]. OT is produced almost solely in the hypothalamic supraoptic (SON) and paraventricular (PVN) nuclei, and is released either to the periphery via the pituitary gland, or within the brain via multiple pathways [3]. All central and peripheral actions of OT are mediated through one oxytocin receptor (OXTR), which is the product of a single gene [4], [5], [6]. Activation of the OXTR promotes coupling to two alternative G proteins, Gq and Gi/o both of which lead to activation of proteins in the MAP kinase signaling pathway such as ERK 1/2 and p38 [7], [8], [9].

Transcription of the *rodent Oxtr*was studied in brain and peripheral tissues where it was found to be partly regulated by estrogens [10], [11], [12], [13], [14]. For example, in parallel to the estrogen levels, *Oxtr* peaks in both the uterus and mammary glands before and during labor. However, post partum its levels drop significantly in the uterus, but remain high in the mammary glands [15], [16]. Furthermore, rats that underwent gonadectomy, showed a positive response to external estrogen in the uterus [17], but not in the mammary gland [18], suggesting distinct regulatory mechanisms in these two tissues. The mechanism by which estrogen regulates *Oxtr* transcription is elusive, partly because the *Oxtr* promoter of several mammalian species, including that of humans, lacks a palindromic estrogen response element (ERE) [1], [4], [5], [6], [19]. Importantly, while *Oxtr* expression can be upregulated by estrogen in tissues, multiple attempts to obtain a similar effect in cultured cells were unsuccessful [12].

DNA methylation is a major epigenetic mechanism that regulates gene transcription [20], [21], [22]. It involves direct chemical modification of a cytosine, immediately followed by a guanine (CpG). These CpG dinucleotides are highly underrepresented in the genome, and often occur in small clusters known as CpG islands [23]. Hypermethylation of CpG sites in the vicinity of genes is often associated with suppression of transcription [20], [21]. In the present study, we hypothesized that transcription of the mouse *Oxtr* is regulated by DNA methylation of specific sites in its promoter, in a tissue-specific manner.

## Materials and Methods

### Ethics statement

All experimental protocols were approved by the Animal Care and Use Committee of the University of Haifa.

### DNA constructs

To construct the *Unmodified* vector, the *CMV* promoter of the *pEYFP-N1* vector (Clontech Laboratories, Mountain View, CA) was replaced by the *Oxtr* minimal promoter [24], amplified (see Table 1 for primers) from C57BL/6 mouse genomic DNA, using AseI/BamHI digestion. CpG sites 1 and 7 were mutated (C to A) by a PCR-based method [25] to produce the *Mut 1* and *Mut 7* vectors, respectively. The *Del* construct was created by PCR amplification of the circular *Unmodified* vector excluding the 400 bp amplicon region. In this reaction we used primers designed to contain a non-complementary 5′ sequence consisting of an EcoRI site (Table 1). PCR amplifications were performed using the KAPAHiFi™ DNA Polymerase (Kapa Biosystems, Woburn, MA) as follows: 95°C for 2 min, 17 cycles of 98°C for 30 s, 60°C for 30 s, 72°C for 40 s and 72°C for 5 min. PCR products were then treated with DpnI to eliminate the template DNA and with EcoRI to produce sticky ends. The digested PCR products were then ligated and transformed into DH5α *E.coli*.

**Table 1:** PCR primer sets.

Primers for amplification of:	Sequence	T_A_
*Unmodified* vector	F: 5′-GGCCATTAATTTAATTAACCTTATTTTACAGATGG-3′ R: 5′-GGAAGGATCCACTAGTAGATTGGGAAAGCTGCTG-3′	59°C, 30 s
*Mut 1* vector	F: 5′-GTTCCTTTTGGTCCTAGGTCATTGTAAATCAG-3′ R: 5′-CTGATTTACAATGACCTAGGACCAAAAGGAAC-3′	60°C, 30 s
*Mut 7* vector	F: 5′-GGACTGGGGGTGGGGAGGGGATACAGGGTGTG-3′ R: 5′-CACACCCTGTATCCCCTCCCCACCCCCAGTCC-3′	60°C, 30 s
*Del* vector	F: 5′-GGGAATTCCTCTCAAGGCTGGTGGGAG-3′ R: 5′-GGGAATTCGCTCTCTAACGTGATTA-3′	60°C. 30 s
*Oxtr* mRNA	F: 5′-GTGCAGATGTGGAGCGTCT-3′ R: 5′-GTTGAGGCTGGCCAAGAG-3′	60°C, 30 s
*Hprt1* mRNA	F: 5′-GGGATTTGAATCACGTTTGTG-3′ R: 5′-TTGCGCTCATCTTAGGCTTT-3′	60°C, 30 s
Bisulfite treated *Oxtr* First PCR round	5′- TTGGTTTTTTTTGTTTTTTTTG-3′ 5′- AAAAAATATCTACCCCTCCCA-3′	54°C, 30 s
Bisulfite treated *Oxtr* Second PCR round (Nested PCR)	5′- TTGGTTTTTTTTGTTTTTTTTG-3′ 5′- CCACCAACCTTAAAAAAAATCT-3′	54°C, 30 s

T_A_, Temperatures and time applied for annealing.

### *In vitro* methylation

20 µg of the examined vectors were methylated *in vitro* in 200 µl reaction mixture, containing 40 U of CpG methyltransferase (*M. Sss*I) and 160 mM *S*-adenosyl methionine (SAM; New England Biolabs, Ipswich, MA) at 37°C for 3 h, with subsequent inactivation of enzyme at 65°C for 20 min. Due to its low stability at 37°C, SAM was added again to the reaction mixture after 1.5 h. Mock-methylation reactions were performed in the absence of *M. Sss*I. Following the enzymatic reaction, the different vectors were phenol/chloroform extracted. The efficiency of methylation for each of the constructs was confirmed by the methylation-sensitive restriction enzyme FauI (New England Biolabs, Ipswich, MA) that has one recognition site in the analyzed region and no sites within the sequence of the *pEYFP-N1* vector.

### Cell cultures and treatment

4T1 mouse mammary carcinoma and GT1-7 immortalized hypothalamic cell lines were kindly provided by Dr. Alon Chen (Weizmann Institute, Israel) and grown in RPMI-1640 and Dulbecco's modified essential medium (DMEM) media, respectively, both supplemented with 10% fetal calf serum and penicillin/streptomycin. Cells were maintained at 37°C humidified incubator with 5% CO_2_. Demethylation experiments were performed in 6-well dishes at a density of 4×10^5^ cells per well. Cells were incubated with 5-Aza-2′-deoxycytidine (5-AzaC, Sigma, St. Louis, MO) at the indicated concentrations for two days, during which medium was replaced daily. ERK activation was measured in GT1-7 cells following overnight starvation, and subsequent stimulation with 1 µM OT for 10 min. Samples were then harvested in RIPA/SDS buffer (50 mM Tris pH 8, 150 mM NaCl, 5 mM EDTA, 1% v/v NP-40, 0.5% w/v deoxycholic acid, 0.1% w/v SDS, 10 mM NaF, 0.1 mM PMSF and Complete Protease Inhibitor cocktail tablets (Roche, Basel, Switzerland). Protein concentrations were determined with the Bradford Assay (Bio-Rad Laboratories, Hercules, CA) and equal amounts of total protein were separated by SDS-PAGE, transferred onto nitrocellulose membranes. Phosphorylation of ERK1/2 was detected by protein immunoblotting using a 1∶1000 dilution of rabbit polyclonal phospho-specific ERK antibody, followed by probing for mouse monoclonal total ERK antibody (Santa Cruz Biotechnologies, Santa Cruz, CA). Proteins were visualized by a Chemiluminescemnce Detection Kit for HRP (EZ ECL, Biological Industries, Beit Haemek, Israel) and quantified using a CCD camera and Quantity One software (XRS, Bio Rad).

### Transfection and reporter gene expression

4×10^5^ GT1-7 cells were seeded in 6-well plates at 80–90% confluence and allowed to grow overnight. Transfection of the different constructs was carried out using the PolyJet™ transfection reagent (SignaGen Laboratories, Rochville, MD), according to the manufacturer's instructions. The FCW_tdTomato vector, which expresses the tdTomato red fluorescence protein under the CMV promoter, was co-transfected with the *EYFP* vectors to control for transfection efficiency and was used to average the levels of EYFP florescence over all tdTomato-expressing cells.

48 h after transfection, the number and fluorescence levels of tdtomato and EYFP expressing cells were analyzed using flow cytometry (FACS Calibur, Becton Dickinson, Franklin Lakes, NJ).

### Animals and tissue processing

Female C57BL/6 mice, 12–16 weeks of age, weighing 20–25 g, were used in all experiments. Animals were housed under diurnal lighting conditions and allowed food and tap water *ad libitum*. Animals were sacrificed following light anesthesia (Isoflurane, Abbott Laboratories, Abbott Park, IL) by cervical dislocation, and tissues were harvested and immediately frozen in liquid nitrogen and then stored at −80°C pending analysis.

### RNA isolation and qPCR

Total RNA was isolated using the TRI Reagent® kit (Sigma, St. Louis, MO) according to manufacturer's protocol. 1 µg and 400 ng of total RNA from cell lines and mouse tissues, respectively, underwent reverse transcription to cDNA using the High Capacity RNA to cDNA kit (Applied Biosystems, Foster City, CA). Transcription of the *Oxtr* was assessed by Real-Time PCR using primers described in Table 1, carried out in triplicates using a 7500 Real-time PCR system (Applied Biosystems) using fluorescent SYBR Green technology. The hypoxanthine-guanine phosphoribosyltransferase (*Hprt*) gene was used as endogenous control. cDNA equivalent to 15 ng of total RNA was added to a 20 µl total reaction mixture with sequence specific primers at a final concentration of 250 nM.

### DNA extraction, bisulfite treatment and sequencing

DNA exaction was carried out using the DNeasy Blood & Tissue Kit (Qiagen, Valencia, CA), according to the manufacturer's instructions. 200–400 ng DNA was subjected to a sodium bisulfite treatment using the EpiTect Bisulfite Kit (Qiagen). The *Oxtr* promoter amplicon was amplified from the bisulfite modified DNA by two rounds of PCR amplification using a hemi-nested primer approach (see Table 1 for primers). In the first round, 3 µl of the modified DNA were amplified in a total volume of 20 µl. Each bisulfite treated sample was amplified in triplicates, which were then pooled together, and 5 µl were subjected to a second round of PCR in a 50 µl total reaction volume. The pooled PCR products of each sample were separated on a 1.5% agarose gel, extracted from the gel with the Illustra GFX™ PCR DNA and Gel Band Purification Kit (GE Healthcare Life Sciences, Piscataway, NJ) and ligated into the pGEM-T-easy vector (Promega, Madison, WI). 2 µl ligation product of each sample were then transformed into DH5α *E.coli*. At least 20 independent recombinant clones per sample were sequenced at by the NINDS DNA Sequencing Facility (Bethesda, MD) and the methylation data was compiled using the BISMA software (http://biochem.jacobs-university.de/BDPC/BISMA/). The Lower threshold conversion rate was set to 90%, while all the other analysis parameters were set to the BISMA default values. CpG island location was determined using Methyl Primer Express software (Applied Biosystems).

### Statistical analysis

Data are expressed as means ± SEM. All statistical tests, as detailed in the figure legends, were performed using SPSS 19.0 (IBM, Armonk, NY) following a normality check by Kolmogorov-Smirnov test. *Post-hoc* Tuckey test was used throughout. All statistical analyses are detailed in the figure legends.

## Results

### Negative correlation between methylation of the Oxtr promoter and its expression

**Figure 1:**
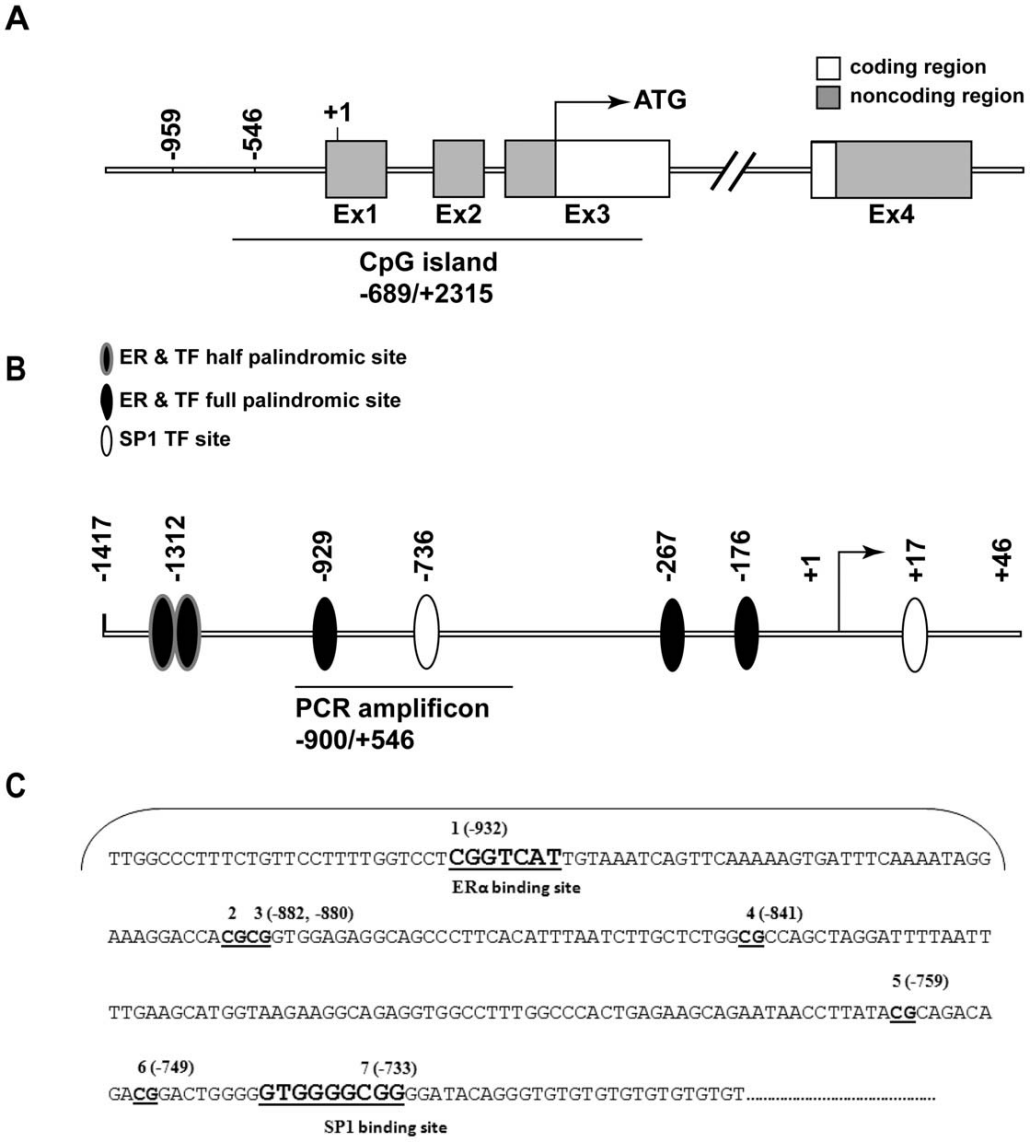
The general structure of the mouse *Oxtr* gene and its promoter. **A**) A schematic depiction of the mouse *Oxtr* gene, including the CpG island that extends from the promoter into the coding region at the third exon. **B**) A schematic depiction of the EREs and SP1 binding sites in the minimal gene promoter, and the relative location of the PCR amplicon between positions −956 and −541. **C**) The sequence of the PCR amplicon, including the positions of the seven CpG sites. The ERE harboring CpG site 1 and the SP1 binding sequence harboring CpG site 7 are underlined.

The mouse *Oxtr* gene is comprised of four exons and three introns [24]. A CpG island extends from the promoter region (−689) into the coding sequence on the third exon (+2315) (Fig. 1A). The minimal promoter of the gene contains several half-palindromic estrogen receptor elements (half EREs), a palindromic ERE and two SP1 sites (Fig. 1B). We focused our analysis of DNA methylation on a ∼400 bp region within the promoter (herein termed *amplicon*), which contains seven CpG sites. The first CpG site (−932) is located within a half-ERE binding sequence, and the last one, (−733) resides within one of the SP1 binding sequences (Fig. 1C). Both of these transcription factors have been implicated in *Oxtr* transcription regulation [26]. Thus, DNA methylation in these sites may regulate *Oxtr* transcription by modulating the binding of the relevant transcription factors to the gene promoter.

**Figure 2:**
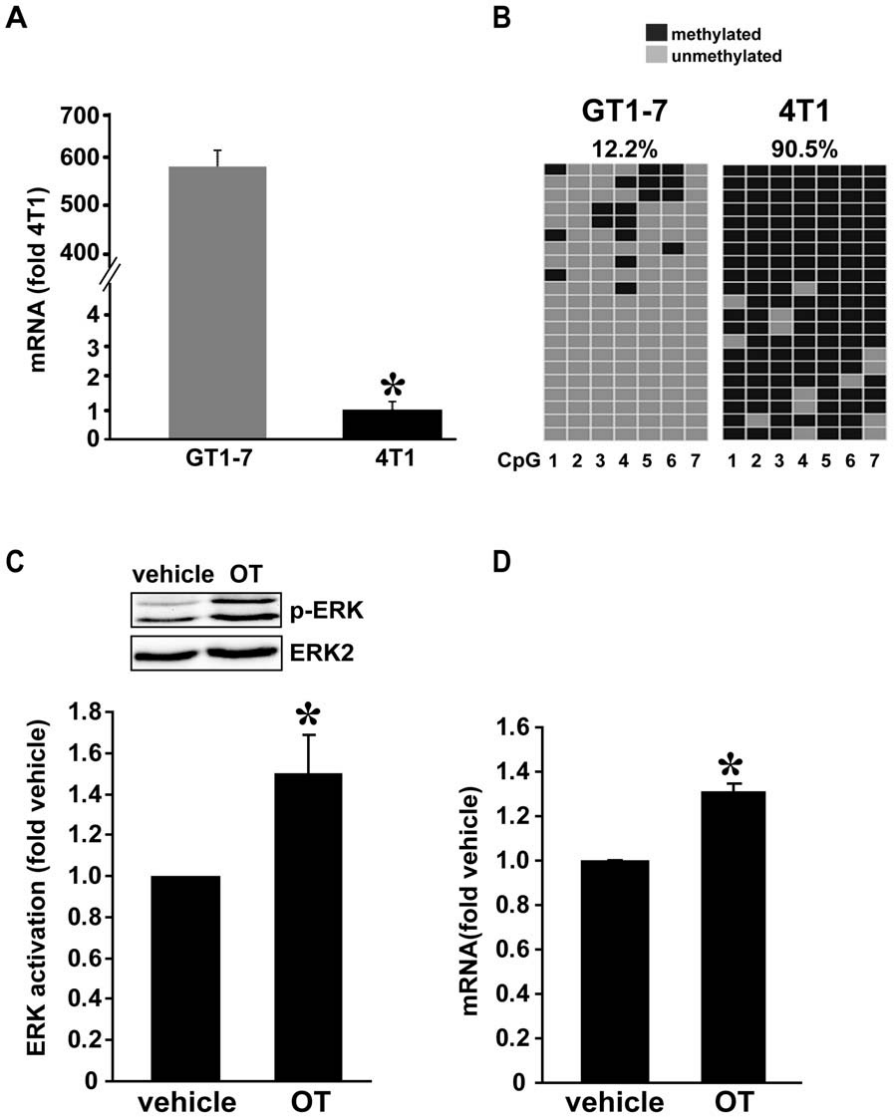
*Oxtr* mRNA levels correlate with *Oxtr* promoter methylation in cell lines. **A**) The relative mRNA levels in GT1-7 are significantly higher than in 4T1 cells. **B**) Methylation of the seven CpG sites is higher in 4T1 cells than in GT1-7 cells. Each row represents a single clone and each column represents one of the seven CpG sites. The total percentage of methylation was calculated from the fraction of black spots (methylated CpG sites). **C**) Representative gel showing greater ERK phosphorylation in GT1-7 cells stimulated with 1 µM OT for 10 min compared vehicle-treated cells Graph is summary of three independent experiments. **D**) Quantities of *Oxtr* mRNA in GT1-7 cells following 24 h treatment with 1 µM OT. An OT-stimulated increase in *Oxtr* mRNA was documented in three independent experiments. **Statistics** (t-test): **A**) *p<0.001, n = 4. **C**) *p<0.05, n = 3. **D**) *p<0.01 n = 3.

To test whether *Oxtr* transcription is associated with DNA methylation of its promoter, we first used quantitative real-time PCR (qPCR) to compare *Oxtr* mRNA levels in different mouse-derived cell lines. As shown in Fig. 2A, the GNRH-releasing immortalized GT1-7 neurons express high levels of *Oxtr* mRNA, ∼600-fold higher than the mammary gland-derived 4T1 carcinoma cells. We then used the bisulfite sequencing method to analyze the methylation level of the *Oxtr* promoter in both cell lines. As depicted in Fig. 2B, the levels of DNA methylation in all seven CpG sites were about 10 fold higher in 4T1 cells compared to GT1-7 cells. The strong negative correlation between the methylation level of the *Oxtr* promoter and mRNA abundance is consistent with a role of DNA methylation in the transcription regulation of the *Oxtr* gene. In order to verify that the high *Oxtr* mRNA levels in GT1-7 cells result in a functional Oxtr protein, we stimulated these cells with OT and measured changes in ERK 1/2 phosphorylation. As shown in Fig. 2C, 10 minutes of OT stimulation caused a marked increase in phosphorylated ERK. Furthermore, exposure of the cells to OT caused a specific elevation in *Oxtr* mRNA levels (Fig. 2D). Together these results link *Oxtr* promoter methylation to gene transcription and protein expression.

### Demethylation enhances Oxtr mRNA expression in 4T1 cells

**Figure 3:**
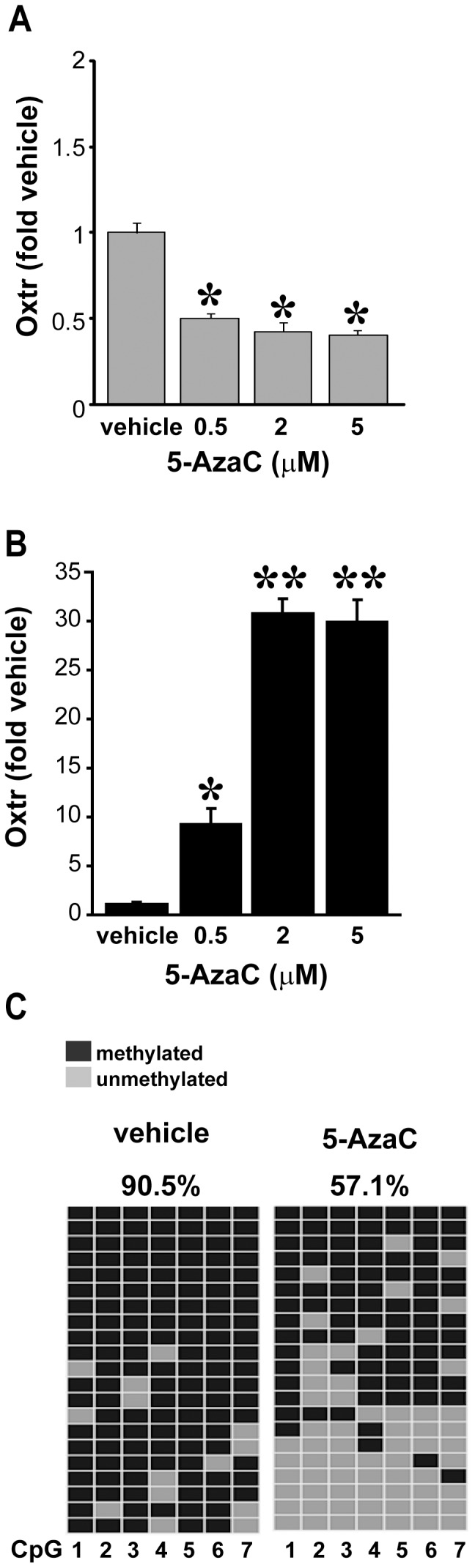
Demethylation induces *Oxtr* expression in 4T1 cells. **A**) In GT1-7 cells, which normally show high *Oxtr* mRNA levels and low methylation, A two-day treatment with 5-AzaC caused a slight reduction in *Oxtr* mRNA levels **B**)Conversely, treatment of 4T1 cells with 5-AzaC caused a robust dose-dependent increase in *Oxtr* mRNA levels.. **C**) 5-AzaC treatment (2 µM) of 4T1 cells caused a significant reduction in the methylation of the *Oxtr* promoter (compared to vehicle samples, also shown in Fig. 2B) across all seven CpG sites of the amplicon. **Statistics**: **A**) One-way ANOVA F_(3)_ = 90.1, *p*<0.001, * differ from vehicle-treated, † differ from 0.5 µM, *p*<0.05, n = 5. **B**) One-way ANOVA F_(3)_ = 39.6, *p*<0.001, * differ from vehicle-treated, *p*<0.01, n = 5.

To further establish a connection between methylation of the *Oxtr* promoter and the mRNA levels of *Oxtr*, we treated both cell lines with the demethylating agent 5-Aza-2′-deoxycytidine (5-AzaC) [27]. GT1-7 cells, which display very low methylation and very high *Oxtr* expression to begin with, responded to 5-AzaC treatment with a mild reduction in *Oxtr* mRNA levels (Fig. 3A). The reason for this slight decrease is unclear and may be caused by a general shift in the balance of gene expression in the cells. Conversely, treatment of 4T1 cells with 5-AzaC caused a dose-dependent increase in *Oxtr* mRNA levels. A maximal ∼30 fold increase in mRNA levels was obtained at a concentration of 2 µM 5-AzaC, and a further increase in dose was ineffective (Fig. 3B).The increase in *Oxtr* mRNA measured in 4T1 cells was accompanied by a significant reduction in the methylation of the *Oxtr* promoter (Fig. 3C). These results strongly support a relationship between the level of *Oxtr* promoter methylation and the transcription of the gene.

### *In vitro* methylation of the *Oxtr* promoter reduces reporter gene expression

**Figure 4:**
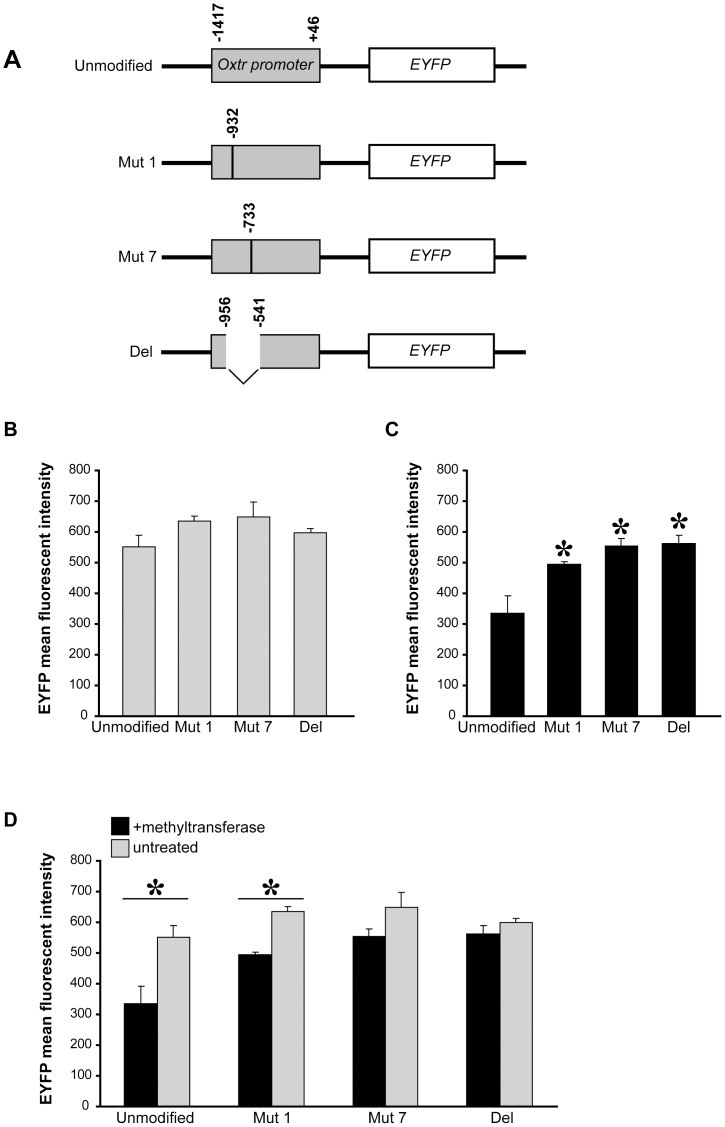
Methylation of specific CpG sites in the *Oxtr* minimal promoter inhibits transcription. **A**) A schematic depiction of the distinct *Oxtr* promoter/*EYFP* constructs used; An *EYFP* gene was coupled to a minimal promoter (positions −1417 to +46) of the mouse *Oxtr* gene (*Unmodified*). This construct was modified by a C to A mutation at CpG sites 1 (*Mut 1*) or site 7 (*Mut 7*) or by deleting the ∼400 bp amplicon region (*Del*). **B,C**) EYFP mean fluorescence intensity measured in GT1-7 cells that were transfected with either (**B**) untraeatd or (**C**) methyltransferase-treated *Oxtr* promoter/*EYFP* constructs. No significant differences were found among the untreated plasmids whereas a highly significant difference was found among the methyltransferase-treated plasmids. **D**) A comparison between the treatedand untreated versions of all plasmids. Highly significant differences were found only in the cases of the *Unmodified* and *Mut 1* plasmids. **Statistics**: **B**) one-way ANOVA F_(3)_ = 2.32, *p*>0.1. **C**) - one-way ANOVA F_(3)_ = 9.4, *p*<0.01, * differ from *Unmodified*, *p*<0.05, n = 5. **D**) t-test, *p<0.05/4.

5-AzaC treatment can affect *Oxtr* expression either directly through modifying DNA methylation of the gene promoter, or indirectly by modulating other mechanisms of transcriptional regulation. In order to discriminate between these possibilities, we designed a reporter gene assay, where the *Oxtr* minimal promoter was introduced upstream to the *EYFP* reporter gene (*Oxtr promoter/EYFP*) to create the *Unmodified* construct. We then created modified versions of this plasmid by introducing A to C mutations in CpG sites 1 (*Mut 1*) or 7 (*Mut 7*), located within a half ERE and the SP1 binding site, respectively. We focused our analysis on these two sites since they are located within putative binding sites of transcription factors known to regulate OXTR expression. We also deleted the whole ∼400 bp amplicon region from the *Unmodified* plasmid to create the *Del* construct (Fig. 4A). We then used the *Sss*I CpG methyltransferase to methylate these constructs in order to check whether DNA methylation regulates *EYFP* expression. To that end, GT1-7 cells were transfected with either the methyltransferase -treated or untreated version of each construct. To control for transfection efficiency, cells were co-transfected with a plasmid containing tdTomato coupled to CMV promoter, and the levels of EYFP florescence were averaged over all tdTomato-expressing cells. A comparison between the untreated versions of the different constructs revealed that none of the mutations by themselves chang EYFP expression (Fig 4B). In contrast, when methyltransferase-treated plasmids were used, even the single methylation-preventing mutation of either CpG site 1 or CpG site 7 caused a significant elevation in EYFP expression, as well as the deletion of the whole amplicon (Fig. 4C). When the mean fluorescence values of the treated and untreated versions of each construct were compared, only the *Unmodified* and *Mut 1* constructs displayed significant differences (Fig 4D). Importantly, the mutation at site 7, which is located within the SP1 binding sequence, was as effective as the deletion of the whole amplicon in abolishing the effect of methylation, suggesting that it is the most effective site of methylation in this region. Together these results indicate a direct effect of methylation of the *Oxtr* promoter on its transcription-inducing activity in cell lines.

### Methylation of the *Oxtr* promoter is modified under different physiological conditions *in vivo*

**Figure 5:**
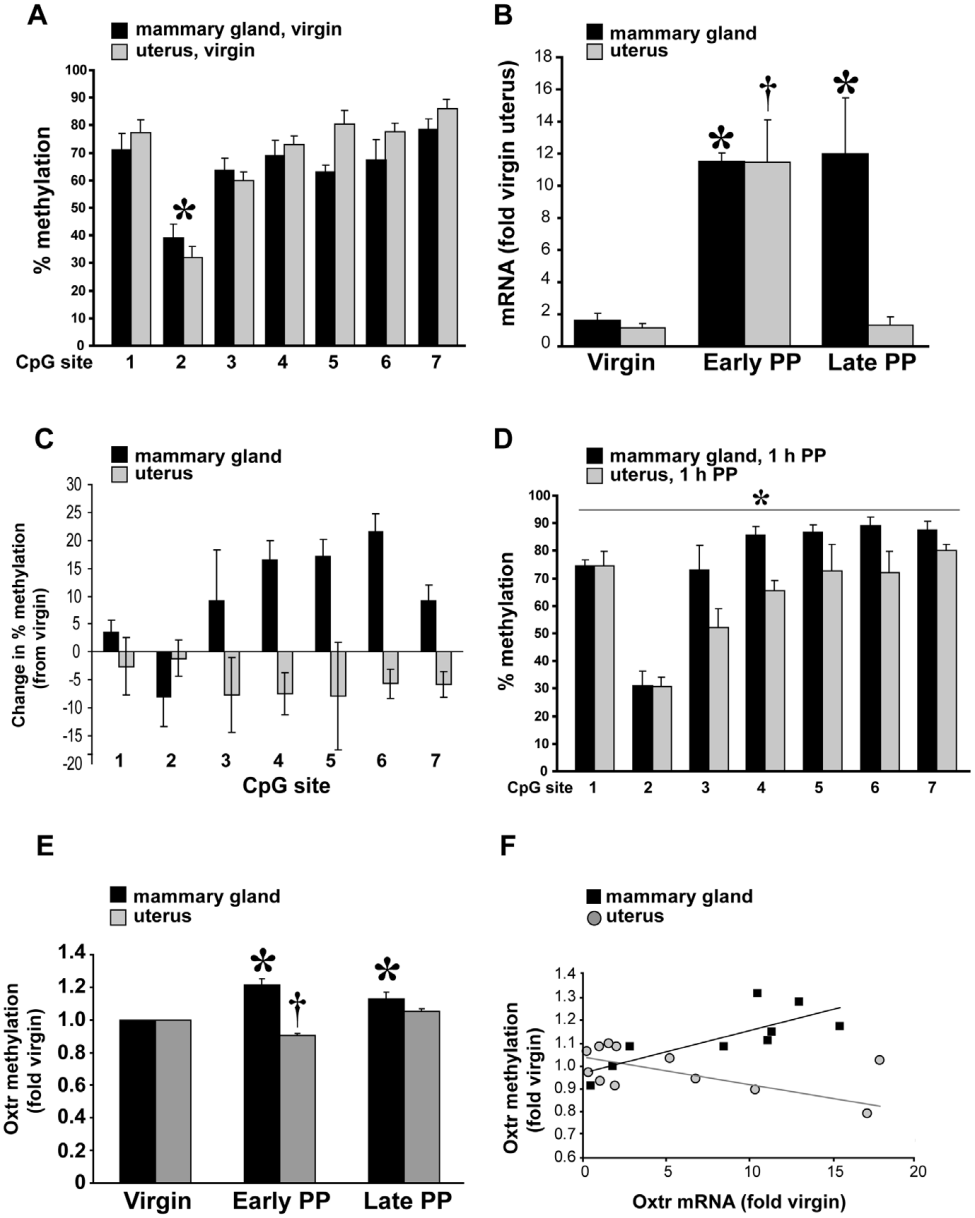
*Oxtr* promoter methylation changes *in vivo*. **A**) The methylation pattern of the *Oxtr* promoter shows no significant difference between the mammary glands (black bars, n = 3) and uterus (gray bars, n = 4) of virgin mice. Nevertheless, a significant difference in methylation was found between CpG sites among all animals. **B**) *Oxtr* mRNA levels in the mammary glands (black bars, n = 4) and uterus (gray bars, n = 5) are significantly elevated 1 h following parturition (early postpartum). Whereas in the uterus mRNA levels dropto background 24 h later (late postpartum, n = 3), they remain high in the mammary glands (n = 2). **C**) The mean difference in the methylation of the seven examined CpG sites, between early postpartum and virgin females. A general increase is observed in the mammary glands while a general decrease is evident in the uterus. **D**) In contrast to virgin mice a statistically significant difference in *Oxtr* promoter methylation is observed between the mammary glands (black bars) and uterus (gray bars) of early post-partum mice. These differences are found in CpG sites 3–7. **E**) The mean relative methylation level of the *Oxtr* promoter, averaged over CpG sites 3–7, is decreased in the uterus in early postpartum and returns to baseline 24 h later. In contrast, in the mammary glands it remains high during late postpartum. **F**) The mean relative methylation level of the *Oxtr* promoter, plotted as a function of the *Oxtr* expression in all animals. A statistically significant positive correlation is seen in the mammary glands (black rectangles), compared to a negative correlation in the case of the uterus (gray circles). **Statistics**: **A**) Between conditions - repeated two-way ANOVA F_(6,30)_, between groups *p*>0.2, interaction between factors *p*>0.4. Between CpG sites - repeated one-way ANOVA F_(6)_ = 20.9, *p*<0.001, * differ from all others *p*<0.05. **B**) Uterus - one-way ANOVA F_(2)_ = 10.1, *p*<0.005, * and † differ from virgins, *p*<0.01. Mammary glands - one-way ANOVA F_(2)_ = 19.6, *p*<0.005, * differ from virgins *p*<0.01. **D**) Repeated one-way ANOVA F(1) = 6.0, *p*<0.05. **E**) Uterus - one-way ANOVA F_(2)_ = 50.6, *p*<0.001, * differ virgins *p*<0.01. Mammary glands - one-way ANOVA F_(2)_ = 11.4, *p*<0.005, † differ from all others *p*<0.01). **F**) Mammary glands - Pearson correlation, *p*<0.01, R^2^ = 0.61. Uterus - Pearson correlation, *p*<0.05, R^2^ = 0.48.

After confirming the effect of DNA methylation on the transcription of the *Oxtr* gene *in vitro*, we used an *in vivo* model where *Oxtr* expression was previously shown to be regulated by hormonal modulation [12], [17], [18]. First, we compared the methylation of the *Oxtr* promoter in the uterus and mammary glands of virgin mice. As depicted in Fig. 5A, no significant differences were observed between the two tissues. Interestingly, in both cases we found significantly less methylation at CpG site 2, a phenomenon that differs profoundly from the pattern observed in cell lines (Fig. 3D). Next, we monitored the methylation and transcription of the *Oxtr* 1 h and 24 h after parturition (early and late post-partum, respectively). As shown in Fig. 5B, *Oxtr* mRNA levels in the uterus increased by about 10-fold in early post-partum compared to virgin mice and returned to baseline in late post-partum. On the other hand, the *Oxtr* mRNA levels in the mammary glands increased to the same extent as the uterus in early post-partum but remained high 24 hours after parturition. Analysis of the *Oxtr* promoter methylation in early post-partum mice revealed opposite changes from virgin mice in both tissues, i.e., methylation is increased in the mammary glands and decreased in the uterus (Fig. 5C). Accordingly, in early post-partum mice there is a significant difference in the pattern of *Oxtr* promoter methylation between the mammary glands and the uterus (Fig. 5D).

The differences between the methylation patterns of virgin and early post-partum mice appear to be CpG-site specific and localized to CpG sites 3–7. Further analysis of the mean methylation averaged over CpG sites 3–7 in the uterus showed a mirror image of the expression levels: mean methylation was reduced during early post-partum and returned to baseline at late post-partum (Fig. 5E). An opposite relationship was observed in the mammary glands, where methylation was elevated both in early and late post-partum mice (Fig. 5E). Finally, examination of the relationship between *Oxtr* mRNA and mean *Oxtr* promoter methylation at CpG sites 3–7 across all animals revealed a negative correlation in the uterus, and a positive one in the mammary glands (Fig. 5F). Together these data show that methylation of the *Oxtr* gene promoter is modified along with its expression levels in a tissue-specific manner *in vivo* under different physiological conditions.

## Discussion

In the present study, we explored for the first time the pattern of methylation in the promoter of the murine *Oxtr* gene and its association with the gene expression *in vitro* and *in vivo*. We used two mouse-derived cells lines, the GnRH-releasing GT1-7 immortalized neurons, and the mammary gland-derived 4T1 carcinoma cells to show that DNA methylation has an inhibitory effect on *Oxtr* transcription. We also revealed distinct modes of correlation between changes in *Oxtr* promoter methylation and the gene transcription that take place in the mammary glands and uterus around the time of labor. A suppressive effect of DNA methylation on *Oxtr* expression was found in several studies, which focused on different regions of the human gene [28], [29], [30]. Mizumoto *et al.* showed that a regulatory element within the third intron of the gene is significantly more methylated in OXTR-expressing compared to non-expressing tissues [30]. Kusui *et al.* used human hepatoblastoma cells to show that deletion of a ∼400 bp segment within the CpG island extending from the gene promoter, reduces the suppressive effect of methylation by 62% [29]. This result suggests that the remaining effect (38%) may be attributed to the promoter region itself, which is in accordance with our findings. Hypermethylation of the same segment of the human *OXTR* correlates with low expression levels of the gene in postmortem brain tissues of individuals with autism [28]. Our results differ from these studies by two main aspects: First, we used a mouse model, which is much more accessible for further molecular characterization and manipulation, and second, we analyzed a region of the *Oxtr* promoter enriched with potential methylated transcription factor binding sites. Assuming that multiple regions of the gene are important for the *in vivo* effect on *Oxtr* expression, it is possible that their methylation level is determined in concert by a single regulatory process, which is common to different species and tissues. Alternatively, DNA methylation may act in distinct gene regions in a species- and/or tissue-specific manner. This possibility is supported by our findings of opposite modulation of *Oxtr* promoter methylation between the uterus and mammary glands.

One of the intriguing findings of our study is that CpG sites 1 and 7 are crucial for the overall effect of *Oxtr* promoter methylation on gene expression (Fig. 4). A mutation in CpG site 7, which is located within a putative SP1 binding sequence, was the most significant one, as it was sufficient to abolish the effect of *Oxtr* promoter methylation on transcription, similarly to deletion of the whole amplicon region. This result raises the possibility that methylation of CpG 7 possibly interferes with the binding of SP1, thus affecting the recruitment of SP1-associated regulatory proteins to the promoter of the *Oxtr* gene [31]. A similar phenomenon was observed in other studies, where methylation of SP1 binding sites was shown to attenuate its binding to the promoter regions of several genes such as the human extracellular superoxide dismutase [32], the human α_1d_-adrenergic receptor [33], and the mouse Abcc6 [34] genes, and to impair their cellular or tissue-specific expression.

In addition to CpG site 7, methylation of CpG site 1, located within a half ERE, was also instrumental for the effect of methylation, suggesting a possible interaction between CpG sites 1 and 7. A similar interaction between half ERE and SP1 sites was previously suggested as a mechanism for estrogen –mediated transcription regulation of several dozens of genes including the progesterone receptor, heat-shock protein 27, cathepsin D, uteroglobin and retinoic acid receptor [35], [36]. While these genes are all regulated by estrogen, none of them contain palindromic EREs, but instead their promoters harbor half EREs in close proximity to GC-rich SP1 binding sites. In the cases of the progesterone receptor and cathepsin D, SP1 directly interacts with both the estrogen receptor ERα and its own GC-rich DNA binding site, in a manner which stabilizes the interaction of both transcription factors with the half ERE/SP1 composite site [37], [38], [39]. Similarly, the 5′-flanking region of the *OXTR* gene contains several SP1 binding sites and putative EREs, most of which are half EREs (Fig. 1). Some species are altogether devoid of a palindromic EREs in their *OXTR* gene [4], and even in those that contain them, the palindromic EREs are often unresponsive to estrogen [12]. This may suggest that the estrogen effect on *OXTR* expression is not mediated through a classical interaction with its binding site. Indeed in the ovine species, an indirect effect of estrogen on *Oxtr* was found to be mediated by SP1 elements [26]. Our results further support this notion by proposing that DNA methylation may affect the interaction between SP1, estrogen receptor and their binding sites in the *Oxtr* promoter. Further investigations are warranted in order to prove that DNA methylation in these sites alters binding to their transcription factors.

The data presented in this study, shows a profound difference between methylation pattern of the *Oxtr* promoter in cell lines and animal tissues. Specifically we show that independent of expression levels, the pattern of methylation across all CpG sites in the cell lines is uniform. Conversely, the uterus and mammary glands present CpG site-specific methylation, with significantly lower methylation at CpG site 2. An area where methylation is significantly lower than its surroundings may be important for certain chromatin structures that promote an interaction between relatively distant elements (∼200 bp apart) such as the SP1 and half ERE sites on the *Oxtr* promoter. The differences we found between methylation in cell lines and tissues may explain why in cultured cell lines, where this CpG specific methylation pattern is absent, there is no effect of estrogen on *Oxtr* expression [5]. This idea is further supported by the observation that bovine endometrial epithelial cells respond to estrogen while in tissue explants, [40], but loose responsiveness when transformed into cell cultures [41].

Our results show for the first time that *Oxtr* promoter methylation varies in relevant tissues, such as the uterus and mammary glands, during and after labor, within a time frame of 24 h. This observation may contradict the common dogma that DNA methylation is a process which occurs during differentiation and remains stable throughout life [22], [23]. Yet, several studies support a rapid and dynamic process of methylation/demethylation, including in memory formation [42] and responses to external stimuli [43]. In fact, the methylation of the OXTR itself was documented to increase within ten minutes of psycho-social stress [44]. The magnitude of our observed changes *in vivo* are smaller (<30%) than the ones we found in cell lines. This difference may be due to the multiple cellular populations comprising the tissue samples, of which only a fraction express the *Oxtr*. In addition, these changes correlate with the well-documented alterations in *Oxtr* expression in these tissues; transient increases in *Oxtr* levels in the uterus before labor, and an increase in the mammary glands that persists post-partum [13], [15], [18]. Nonetheless, the relationship between *Oxtr* expression and methylation is opposite between both tissues. The changes observed in the uterus, are in line with the known inhibitory effect of methylation on gene expression as we showed in cell lines. In contrast, in mammary tissue, both methylation and expression levels of *Oxtr* are high in post partum mice, suggesting an alternative mechanism for regulation which might also be modulated by methylation. It should be noted that unlike the uterus, *Oxtr* expression in the mammary glands is not sensitive to induction by estrogen [18].

Together the data presented in this study shows both *in vitro* and *in vivo* that transcription of the mouse *Oxtr* is directly regulated by methylation of its promoter, in a tissue and physiological condition-specific manner. This mechanism may serve to modulate the expression of the *Oxtr* in the brain, where it has been shown to have a profound effect on mammalian social behavior and to be associated with pathologies such as autism [28], [45], [46], [47], [48], [49]. To our knowledge, this is the first study to explore DNA-methylation-mediated epigenetic regulation of the *Oxtr* in the mouse. It provides an important tool to investigate environmental influences on *Oxtr* expression and their effect on oxytocin-dependent maternal functions and social behaviors.
